# Influence of Domestic Cooking on Quality, Nutrients and Bioactive Substances of *Undaria pinnatifida*

**DOI:** 10.3390/foods10112786

**Published:** 2021-11-12

**Authors:** Shan Jiang, Yida Wang, Haolin Song, Jiaying Ren, Baomin Zhao, Taihai Zhu, Chenxu Yu, Hang Qi

**Affiliations:** 1 National Engineering Research Center of Seafood, Liaoning Provincial Aquatic Products Deep Processing Technology Research Center, School of Food Science and Technology, Dalian Polytechnic University, Dalian 116034, China; jiangshan_dlpu@163.com (S.J.); wyd8393@163.com (Y.W.); shl6668882021@163.com (H.S.); JiaYing181690@163.com (J.R.); 2 Jiangsu Palarich Food Co., Ltd., Xuzhou 221116, China; steven@palarich.com (B.Z.); zhutaihai@palarich.com (T.Z.); 3 Department of Agricultural and Biosystems Engineering, Iowa State University, Ames, IA 50011, USA; chenxuyu@iastate.edu

**Keywords:** *Undaria pinnatifida*, domestic cooking, bioactive compounds, nutrients, quality

## Abstract

*Undaria pinnatifida* (*UP*) is a brown algae commonly consumed as food in Asian countries. The purpose of this study was to compare the effects of different domestic cooking methods (i.e., air frying (AF), microwaving, and high temperature and pressure (HTP) cooking) on the nutritional and bioactive substances in *UP*, as well as on *UP* color and texture, in order to identify methods to retain beneficial components better. In this study, microwave treatment resulted in better retention of color, polysaccharide (4.17 ± 0.07 mg glucose equivalents (GE)/g dry weight (dw) ), total phenol content (TPC) (1.50 ± 0.0062 mg gallic acid equivalents (GAE)/g dw) as well as chlorophyll a (18.18 ± 0.41 mg/g fresh weight (fw) ) and fucoxanthin (281.78 ± 17.06 μg/g dw). HTP treatment increased the TPC of *UP* (1.69 ± 0.0075 mg GAE/g dw), and AF treatment resulted in a lower loss of total amino acids (2.14 ± 0.15%). Overall, microwave cooking appeared to be the best among the three in producing cooked *UP* with high quality. This study provided a useful guideline in selection of cooking for *UP* which could retain more health-beneficial substances and yield products with better eating qualities to improve human diet.

## 1. Introduction

Seaweeds are simple heterokonts which do not have real differentiation of roots, stems and leaves, nor flowers, fruits and seeds. They are categorized into brown (Phaeophyta) algae, green (Chlorophyta) algae and red (Rhodophyta) algae according to their color, with brown algae being the most consumed, accounting for 66.5% of human consumption worldwide [1]. According to data from Grand View Research, the global commercial seaweed market is expected to grow to USD 22.1 billion by 2024 due to growing demand in the food, pharmaceutical and agricultural sectors [2].

*Undaria pinnatifida* (*UP*) is a marine brown algae, which has long been part of the human diet, especially in Asian countries such as China, Japan and South Korea [3]. The output of *UP* in China reached 225,604 tons in 2020, according to the Chinese Fishery Yearbook 2021. It is well known that *UP* is not only rich in various common nutrients such as minerals, vitamins and dietary fiber, but also contains a variety of bioactive compounds: polyphenols, fucoxanthin, polysaccharides, etc., which have antioxidant, anti-tumor, anti-inflammatory, anti-thrombotic and immunomodulatory properties [4]. For example, fucoxanthin, a kind of carotenoid which accounts for more than 10% of the carotenoids found in nature, is one of the representative active substances in *UP* known for its anti-cancer, anti-obesity and anti-diabetes functions [5]. The chlorophyll in seaweeds has also been shown to have biological activities such as antimutagenic effects [6]. Besides, the dietary fiber content of edible seaweeds can get up to 40%, which is higher than that of common vegetables and could be quite beneficial for human health [7]. For example, the dietary fiber content in the inner part of the onion was 11.6% [8], and 5.6% in three varieties of potato [9].

Cooking methods can directly affect the nutritional properties and the composition of the final ingredients of the food. For example, the medium used in cooking (e.g., water) can affect the concentration of water soluble compounds [10]. The texture and phytochemical properties of plant foods would change during the cooking processes [11]. Murador et al. [12] reported the effects of different cooking techniques on the levels of bioactive compounds in kale and red cabbage. According to Rufián-Henares et al. [13], prolonged cooking may lead to the loss of biologically active compounds. Certainly, cooking such as heat treatment may also increase the availability of bioactive compounds by destroying cell structures in plant foods [14]. In general, all the modifications caused by cooking could have an impact on the physical and chemical properties of the cooked foods.

*UP* is usually cooked before human consumption. However, few existing studies have investigated the effects of cooking on the physicochemical properties of *UP*. In this study, three common home cooking methods, air frying (AF), microwave and high temperature and pressure (HTP), were used to cook *UP*. Therefore, the purposes of this study were to: (1) compare the effects of three cooking methods on the nutrients and quality of *UP*; (2) identify better cooking methods to retain bioactive substances and texture, etc. The influences of these methods on the quality (such as color and texture), nutrients and bioactive potential of cooked *UP* were characterized and compared. These findings will provide a basic reference to guide *UP* processing to better prepare it for human consumption.

## 2. Materials and Methods

### 2.1. Materials and Chemicals

The salted *UP* was provided by Dalian Aquatic Breeding Group Co., Ltd. (Dalian, China). The manufacturing process of salted *UP* was as follows: the fresh *UP* was harvested from the ocean, boiled at 90 °C for 30 s and then *UP* was cooled with flowing seawater. The cooled *UP* was partially dehydrated with salt and the salt content in *UP* was controlled at 12.0 ± 1.5%. After trimming away irregular leaves, the *UP* was packed in polyethylene bags and wrapped in cardboard cases. Every box was 10 kg. The salted *UP* in the study was stored in the factory for less than 1 month at 4 °C. After being transported to the laboratory, it was stored at 4 °C until used.

Methanol (analysis grade), acetone (analysis grade, HPLC grade) and hydrochloric acid were supplied by Tianjin Damao Chemical Reagent Factory (Tianjin, China). While methanol (HPLC grade) was bought from Spectrum Chemical Manufacturing Co., Ltd. (Shanghai, China). Folin–Ciocalteu’s phenol reagent (reagent grade) was obtained from Sangon Biotech (Shanghai) Co., Ltd. (Shanghai, China). Anhydrous ethanol (analysis grade) was provided by Tianjin Fuyu Fine Chemical Co., Ltd. (Tianjin, China). Anhydrous sodium carbonate was purchased from Tianjin Guangfu Technology Development Co., Ltd. (Tianjin, China). The fucoxanthin and chlorophyll a standards and gallic acid were purchased from Sigma-Aldrich Co., Ltd. (Shanghai, China). All other reagents used for the analysis were analytical grade.

### 2.2. Undaria Pinnatifida (UP) Preparation and Cooking

Before cooking, salted *UP* samples were soaked in water until they fully stretched out, and then washed 3–4 times with deionized water to remove the salt from the surface. The washed *UP* samples were then cut into pieces of the same size (50 g equal portions). *UP* samples were then prepared with domestic cooking, including AF cooking, microwave cooking and HTP cooking methods.

#### 2.2.1. AF Cooking

50 g of the prepared *UP* were wrapped in 30 cm × 30 cm aluminum foil, then folded into a square of about 20 cm × 20 cm, and cooked in an air fryer (HD 9651, Philips (China) Investment Co. Ltd., Shenzhen, China) at 180 °C for 10 min.

#### 2.2.2. Microwave Cooking

We put 50 g of prepared *UP* into a heat-resistant glass box (KH-8676, Xitianlong Technology Development Co., Ltd., Tianjin, China), and 120 mL of deionized water was added into it, then it was heated in a microwave oven (NE-1753, Panasonic Co., Ltd., Kadoma, Japan) at 2450 MHz-1700 W for 2.5 min.

#### 2.2.3. High Temperature and Pressure (HTP) Cooking

We packed 50 g of prepared *UP* in a glass fresh-keeping box (830 mL, Anhui Deli Daily Glass Co., Ltd., Anhui, China), which was then cooked in an electric pressure cooker (YBD50-90A1(B), Zhangzhou Wanlida Household Electric Appliance Co., Ltd., Zhangzhou, China) with 1 L of deionized water for 10 min (total time of pressurizing and pressure holding of pressure cooker).

Each individual cooking experiment was conducted three times.

### 2.3. Sample Preparation

At the end of cooking, we quickly sealed the *UP* in a self-sealing bag and placed it in ice water to achieve rapid cooling. After quick cooling, part of each of the cooked *UP* was used for analyses of the moisture, texture, color and chlorophyll a content for each cooking method, and the remaining sample was dried for 11 h in a drying oven (PH-070A, Shanghai Yiheng Technology Co., Ltd., Shanghai, China) at 50 °C, then crushed into powder, sieved with a 200-mesh sieve, and stored in a sealed vacuum bag at 4 °C for further analysis. The raw *UP* was analyzed as the control.

### 2.4. Analysis of Water State and Distribution

The states of water in the various *UP* samples were characterized by measuring the transverse relaxation time T_2_ with low-field nuclear magnetic resonance (LF-NMR) analyzer (MesoQMR23-060H, Suzhou (Shanghai) Niumag Electronic Technology Co., Ltd., Shanghai, China). The T_2_ time was determined by the Carr Purcell Meiboom Gill (CPMG) sequence with key parameters: TE (time echo) = 0.5 ms, TW (time waiting) = 4000 ms, NS (number of scan) = 8, NECH (number of echo) = 8000. The parameters were obtained by modifying the method according to Li et al [15].

### 2.5. Determination of Color

The colors of raw and cooked *UP* samples were determined by a chromatimeter (UltraScan PRO, Hunter Lab, Reston, VA, USA). The values of L*, a* and b* were measured at six different places on the same *UP* blade, with each measurement repeated three times. In the International Commission on Illumination (CIE) color system, the negative coordinate of a* represents the intensity of green, and the positive coordinate of b* represents the intensity of yellow. The total color difference (∆E) was calculated according to Rana et al. [16]:

(1) $$∆E_{\mathrm{ab}}=\sqrt{{\left(L^{*}-L_{0}^{*}\right)}^{2}+{\left(a^{*}-a_{0}^{*}\right)}^{2}+{\left(b^{*}-b_{0}^{*}\right)}^{2}}$$

where $L_{0}^{*}$, $a_{0}^{*}$, $b_{0}^{*}$ are the values of the control sample (raw *UP*). The $L^{*}$, $a^{*}$, and $b^{*}$ are the values of samples subject to different cooking methods.

### 2.6. Texture Analysis

Texture attributes are important position in people’s sensory evaluation of the food. The textural properties of raw and cooked *UP* were measured by a texture analyzer (TA-XT plus, Stable Micro Systems Ltd., Vienna, UK) with a 50 mm diameter (P/5) probe. The pre-test, test, and post-test speeds were 2.0, 1.0, and 2.0 mm/s, respectively, which was modified by the previous study [17]. The deformation rate was set to 50% during operation. The stress–strain curve was analyzed and the hardness, springiness, chewiness, cohesion and resilience were calculated automatically by the testing software.

### 2.7. Scanning Electron Microscope (SEM)

A SEM (JSM-7800F, Tokyo, Japan) was used to observe the microstructure of raw and cooked samples to determine the effects of various cooking methods on the structure of *UP*. The method was modified from previous report [18]. Microstructure was examined with a × 500 magnification. The samples were placed in a freeze dryer (Scientz-10ND, Ningbo Scientz Biotechnology Co., Ltd., Ningbo, China) at −80 °C for 48 h, and the freeze-dried *UP* was crisply broken with liquid nitrogen. The effects of various cooking methods on the microstructure of *UP* were imaged from the cross-sections of the broken pieces.

### 2.8. Analysis of Chlorophyll A

The extraction method of chlorophyll a was modified from the previous study [19]. Raw and cooked samples were cut into filaments about 1 cm in length and 1 mm in width, and 2.0 g of samples were subject to extraction with 15 mL of ethanol and acetone 1:1 (*v*/*v*). The sample mixture was sonicated in a 200 W ultrasonic bath (SB-5200D, Ningbo Xinzhi Biotechnology Co., Ltd., Ningbo, China) at room temperature for 20 min. Afterwards, the extract was centrifuged in a centrifuge (L550, Hunan Xiangyi Laboratory Instrument Development Co., Ltd., Changsha, China) at 4000 rpm for 10 min. We took 5 mL of the supernatant and evaporated it to dryness with a rotary evaporator (SY-2000, Shanghai Yarong biochemical instrument factory, Shanghai, China), and redissolved it in acetone (5 mL). It was then filtrated through a syringe filter (0.22 μm) to obtain the chlorophyll a extract.

The chlorophyll a extract was analyzed using Shimadzu series HPLC system equipped with diode array detector (SPD-M20A, Shimadzu Corp., Kyoto, Japan). Labsolutions software (Shimadzu Corp., Kyoto, Japan) was used to monitor and control the pump (LC-20AT), the automatic sampler (SIL-20A, Shimadzu Corp., Kyoto, Japan), the column oven (CT0-20A, Shimadzu Corp., Kyoto, Japan) and the HPLC diode array system. Separation was achieved on the C18 column (5 μm, 4.6 × 150 mm, Shimadzu, Kyoto, Japan) at 30 °C. The mobile phase was 100% methanol (HPLC grade), which was pumped at a flow rate of 0.8 mL/min for a total run time of 20 min. The injection volume was 10 µL. The scanning wavelength range was 200–800 nm, and chlorophyll a was detected at 430 nm. The results were expressed in mg per gram of fresh weight (fw).

### 2.9. Analysis of Fucoxanthin

We weighed 1 g of *UP* powder sample accurately, 10 mL of 80% ethanol was added to the sample, the mixture was placed in an ultrasonic bath at 50 °C for 30 min [20], and it was centrifuged at 4000 rpm for 10 min. We evaporated 5 mL of the supernatant to dryness at 40 °C in rotary evaporator [21]. The extract was redissolved with the same volume of methanol (5 mL) and filtered through a 0.22 μm syringe filter to get the fucoxanthin extract. 

The fucoxanthin extract was also analyzed by Shimadzu HPLC series system. Separation was performed at 40 °C using a C18 column (5 μm, 4.6 × 250 mm, Shimadzu). Methanol (HPLC grade) was used as the mobile phase. The pump flow rate was 0.5 mL/min and the data acquisition time was 10 min. The pump (LC-20AT), autosampler (SIL-20A, Shimadzu Corp., Kyoto, Japan), column oven (CT0-20A, Shimadzu Corp., Kyoto, Japan), etc. operated under the detection and control of Labsolutions software (Shimadzu Corp., Kyoto, Japan). The fucoxanthin was detected by the photodiode array ultraviolet visible light detector (PDA) at 450 nm [22]. The results were expressed in μg per gram of dry weight (dw).

### 2.10. Total Phenol and Polysaccharide

The total phenolic compound (TPC) extract was prepared as previously described with modifications [23]. We extracted 1 g of sample powder with 15 mL of 50% ethanol. The mixture was sonicated for 30 min, shaken at 120 rpm and 50 °C in a water bath (THZ-82, Changzhou Zhiborui Instrument Manufacturing Co., Ltd., Changzhou, China) for 7.5 h. The supernatant was obtained after filtering with Whatman #1 filter paper (Whatman International Co., Ltd., Maidstone, UK), which was the total phenol extract. 

The modified Folin–Ciocalteau method was used to determine the TPC of the extract from the previous step. To 50 µL of the extract, 600 µL of deionized water and 50 µL of Folin–Ciocalteu phenol reagent were added sequentially. We added 20% sodium carbonate solution (150 µL) after 1 min. The mixture was incubated at 37 °C for 1 h, and the absorbance was measured at 760 nm with the microplate reader (Infinite 200, Tecan Austria Co., Ltd., Grodig, Austria). The measured results were compared with the standard curve with gallic acid solution. TPC was expressed as gallic acid equivalents (GAE) per gram of dw.

The dried sample powder and distilled water were mixed (1:90, *w*/*v*) for 30 min and sonicated for 6.5 min for the polysaccharide extraction. The supernatant was obtained by centrifugation for 30 min after 2 h of constant temperature extraction at 50 °C. Three times the volume of ethanol was added to the supernatant, and the crude polysaccharide precipitate was obtained by centrifugation (CR22N, Hitachi Koki Co., Ltd., Tokyo, Japan) at 6000 rpm for 25 min after standing overnight at 4 °C.

After the crude polysaccharide precipitate was naturally evaporated and dried, the collected polysaccharide was mixed with 1 L distilled water. We took 1 mL of the fully dissolved polysaccharide liquid, to which 1 mL of 5% phenol solution and 5 mL of concentrated sulfuric acid were added to measure the polysaccharide content, by the absorbance of the mixture at 490 nm [24]. The polysaccharide content was expressed as glucose equivalents (GE) per gram of dw.

### 2.11. Amino Acid Analysis

Amino acids of each sample were determined by the method of GB5009.124-2016 [25]. Briefly, 500 mg of powdered sample was mixed with 10 mL of 6 M HCl and hydrolyzed in the ampoule at 110 °C for 22 h, the hydrolysis solution was filtered through 0.22 μm filter membrane and filled to 10 mL by 6 M HCl, 3 mL of which was rotary evaporated to dryness at 60 °C and re-dissolved with 0.02 M HCl (3 mL). Determination of amino acids was undertaken by using an ultra-high speed amino acid (LA8080, Hitachl High-Tech Science, Naka, Japan) analyzer after 30 times dilution.

### 2.12. Statistical Analysis

Unless stated otherwise, means and standard deviations of three independent measurements were calculated. Statistical differences were analyzed by one-way analysis of variance (ANOVA) using the IBM SPSS software version 26 (IBM Corporation, Armonk, NY, USA). Significant differences between the detected parameters were compared by means of Duncan’s multiple comparison test at a level of *p* < 0.05.

## 3. Results and Discussion

### 3.1. Color of UP under Different Cooking Methods

Color is closely related to the acceptance of plant foods after cooking [26]. Raw and cooked *UP* were shown in Table 1. The raw sample was used as the control. Observed differences in the effects of different cooking techniques on color coordinates were listed in Table 1. The monochromatic variables a* and b* corresponded to the changes from green to red and from yellow to blue, respectively [27]. In this study, the focus was on the influence of cooking method on the value of a* (i.e., the degree of greenness). The results showed that compared with the control, cooking had a significant effect on the value of a* (*p* < 0.05). As shown in Table 1, regardless of the methods, cooking reduced the value of −a*. AF (29.08 ± 0.03%) and HTP (36.43 ± 0.03%) cooking resulted in a higher loss of greenness (−a*) compared to the microwave group (22.70 ± 0.06%). The phenomenon whereby the microwave group lost greenness less than other groups was consistent with the report by Turkmen et al. [28]. This may be due to the formation of certain chlorophyll derivatives such as chlorophyllides more than pheophytins, which do not cause changes in the chromophore properties and color of their precursors, as explained by Mosquera et al. [29]. Moreover, the use of shorter cooking times in microwave processing also contributed to the less de-coloring. Regarding L* (lightness) and b* (yellowness), the values during different cooking treatments followed similar trends, with a slight increase in the microwaved samples (control to microwaved: 26.48 ± 1.63 to 26.91 ± 1.09 at L*, 44.88 ± 2.64 to 45.58 ± 1.82 at b*) and a decrease in the other two groups relative to the control group, however, they were not affected as significantly as a* values. Akdaş and Bakkalbaşı [30] showed that home cooking did not significantly affect the b* value in kale leaves which remained similar to that of the raw sample, consistent with our results (Table 1). The increase in L* value of the microwaved group may be due to scalding water during cooking replacing air between cells, and water released due to cell membrane rupture which altered its opacity [31]. Regarding the decrease in L* value of AF and HTP groups, similar declines caused by cooking have been observed in other studies [30]. The global color changes (∆E) of cooked samples by different treatments were reported in Table 1. Considering raw *UP* as a comparison, the ∆E value was more affected by HTP treatment (5.90 ± 1.37) than by AF (4.29 ± 1.49) and microwave cooking (3.20 ± 0.66), possibly due to its greater effect on the a* value. According to Severini et al. [32], the AF process tended to remove the air between leaf cells and changed the surface light reflection characteristics of the sample. The data showed that HTP treatment had the greatest effect on the total color change of samples after cooking. Microwave cooking appeared to be able to retain the overall color better than the other two methods as shown in Table 1.

### 3.2. Water States in UP Samples Prepared with Different Cooking Methods Evaluated by Low-Field Nuclear Magnetic Resonance (LF-NMR)

**Figure 1:**
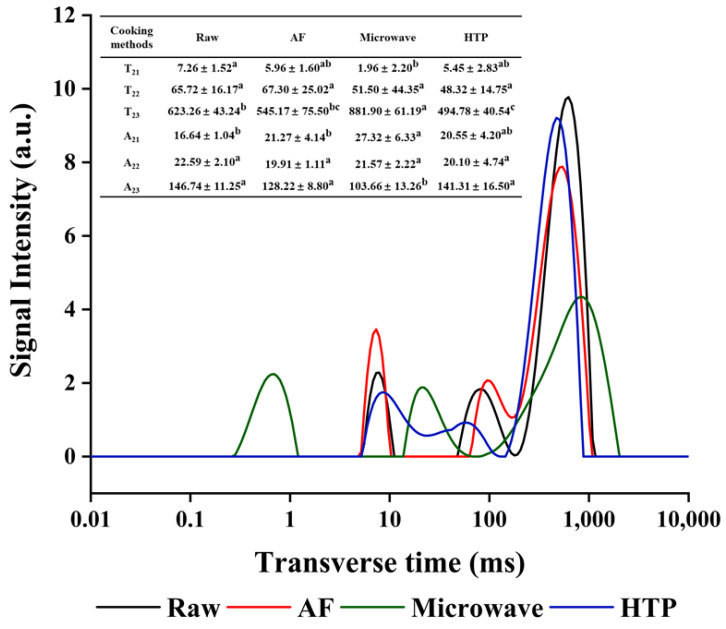
Representative T_2_ relaxation spectra of *UP* treated with different cooking methods. Different letters in the same row for different cooking methods indicate significant differences at (*p* ≤ 0.05).

The water distribution in *UP* prepared with different cooking methods was studied by T_2_ analysis. Figure 1 showed the representative T_2_ relaxation spectrum obtained by multi-exponential fitting of CPMG original data. T_2_ (transverse relaxation time) and A_2_ (total amplitude of T_2_) are two important result indicators of LF-NMR, reflecting the distribution of water in the sample. The value of T_2_ indicates the molecular mobility of the phase water, which is directly proportional to the mobility and inversely proportional to the degree of immobilization. In other words, the larger the T_2_, the more mobile the water in the sample [33]. More specifically, T_2_ band could be further characterized into three peaks of the three different water states, as shown in Figure 1. They represented bound water T_21_ (0.01–10 ms), immobilized water T_22_ (10–100 ms), and free water T_23_ (100–1000 ms), respectively [34]. The three peak intensities are evaluated by peak areas by A_21_, A_22_ and A_23_, which correspond to the content of each water component. T_2_ and A_2_ parameters are shown in Figure 1. Compared to the control, the T_21_ values of the cooked samples fluctuated in the range of 1.96 ± 2.20–5.96 ± 1.60 ms. T_22_ values did not show a significant change (*p* > 0.05). However, T_23_ showed obvious changes, as the microwaved group showed significantly increased (623.26 ± 43.24 to 881.90 ± 61.19 ms) value, suggesting a significant increase in water mobility in free water, which in turn could be more easily removed [33]. For the AF and HTP cooked samples, an apparent reduction in T_23_ relaxation times was observed compared to the control sample, which indicated that the water mobility decreased. In addition, the reduction of A_23_ alongside the increase of A_21_ showed in all cooked samples indicated a general trend of free water being transferred into bound water [33].

### 3.3. Texture Analysis

Texture is an important sensory attribute that affects people’s acceptance of food. Table 2 showed the changes in textural parameters of *UP* samples after cooking, including hardness, springiness, cohesiveness, chewiness and resilience, respectively. It was clear that cohesiveness, hardness, springiness, chewiness and resilience were significantly decreased after cooking in a general trend of control > HTP > AF > microwave. Among them, compared with the control group, the HTP group, AF group and microwaved group showed a decrease in hardness by 25.58 ± 0.03%, 25.85 ± 0.08%, 58.03 ± 0.07%, respectively; in springiness by 9.72 ± 0.07%, 22.20 ± 0.01%, 45.76 ± 0.01%, respectively; in chewiness by 29.18 ± 0.10, 44.27 ± 0.07%, 81.08 ± 0.04%, respectively; in resilience by 5.30 ± 0.06%, 11.29 ± 0.10%, 39.14 ± 0.07%, respectively. Cohesiveness measures the internal force that holds the *UP* together before the compression reached the breaking point for the sample, and its value depends on the sample properties as well as external factors such as temperature and humidity [35]. The cohesiveness of the microwaved group was significantly reduced from the control, while the AF group and the HTP group showed no significant changes. According to previous reports [36], the changes in texture of high-pressure processed vegetables were due to cell rupture, which was conducive to enzymatic and non-enzymatic reactions and pectin methylesterase cleavage. The release of enzymes can be enhanced by pressure. The texture of seaweeds was similarly affected by HTP treatment, and the alginate lyase in seaweeds acted as a pectin methylesterase [37]. Chewiness is defined as the amount of energy required to chew and is related to hardness, elasticity and cohesiveness [38]. It was reasonable, therefore. to see that almost all textural parameters followed the same general trend, as degree of changes from the control due to cooking were HTP group < AF group < microwaved group, with the highest change occurring in the microwaved group. The moisture content and the texture were closely correlated [39], thus, as microwaving cooking brought the most moisture loss (Figure 1), it also had the most significant effect on the textural properties of cooked *UP*.

### 3.4. Scanning Electron Microscopy (SEM)

**Figure 2:**
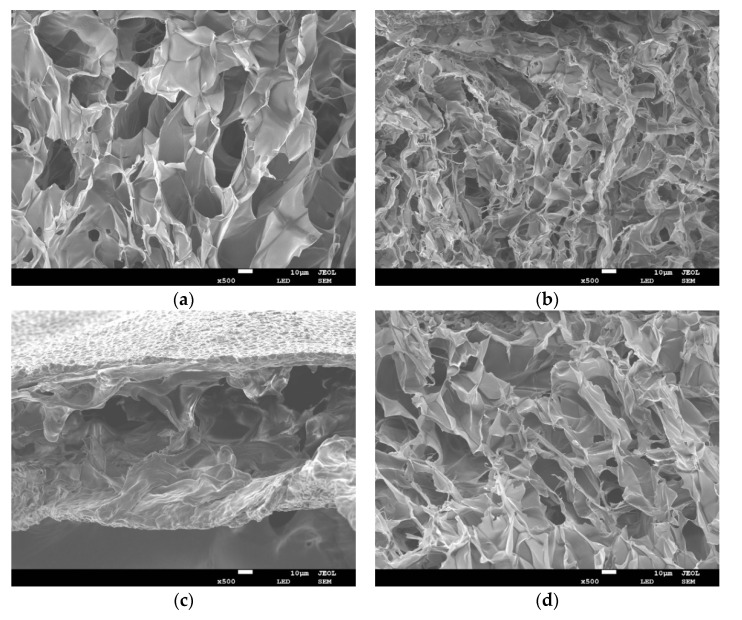
Representative scanning electron microscope (SEM) images of *UP* under different cooking methods (10 μm length scale bar) (**a**) raw; (**b**) AF; (**c**) microwave; (**d**) HTP.

In order to evaluate the effect of different cooking methods on *UP* microstructures, SEM was performed on all samples. Figure 2 shows the representative SEM images of lateral structures obtained from freeze-dried *UP* crisply broken in liquid nitrogen for the control group (i.e., raw, Figure 2a), AF group (Figure 2b), microwave group (Figure 2c), and HTP group (Figure 2d), respectively. As shown, cooking, regardless of the method, significantly altered the internal microstructure of *UP*. In these samples, the tissue structure of *UP* was destroyed. Among them, the reduction of pore size and increase of pore density in the AF group were particularly obvious, this was probably caused by more water evaporation due to the high temperature of 180 °C (Figure 1). In contrast, the pores of the microwaved group were so big. This may be due to the fast heat transfer during microwaving caused water to boil in tissue cells, potentially damaged them [31]. The variation of pores in the HTP group was non-directional (Figure 2d), and the structural collapse was probably due to tissue destruction caused by high temperature and pressure.

### 3.5. Bioactive Substances

#### 3.5.1. Total Phenol Content (TPC) and Polysaccharide Content

It can be seen from Table 3 that the TPCs of the AF group and microwaved group were significantly lower than that of the control group (1.44 ± 0.0038 mg GAE/g dw, 1.50 ± 0.0062 mg GAE/g dw), which indicated that the polyphenols in samples were decomposed during the cooking process. The reduction in TPC in the microwaved group was similar to that reported by Sergio et al. [40] for microwaved asparagus. Oxidation at high temperature, leaching during cooking, and the solubility of polyphenolic compounds in hot water may all contributed to their loss during microwave cooking. The AF group showed the highest loss rate, which was contrary to the results of Salamatullah et al. [41], suggesting that AF under different conditions for different food may lead to different levels of TPC loss. As to the increase in TPC in the HTP group, which was consistent with many studies reporting increase in TPCs in heated/roasted plant samples, could be attributed to the high pressure may break the chemical bonds in the cells and change the distribution and aggregation of phenolic compounds, making the solvent more accessible to the compounds and thus facilitating the extraction [42]. Inactivation of degradative enzymes by heating may also lead to an increase in TPC, which could only be a minor factor considering the opposite trends in TPCs observed in the microwaved and AF groups, where inactivation of enzymes should also have happened.

Polysaccharide contents in all cooked samples were significantly decreased as follows: raw *UP* (4.76 ± 0.20 mg GE/g dw) > microwaved (4.17 ± 0.07 mg GE/g dw) > AF (3.56 ± 0.27 mg GE/g dw) > HTP (2.39 ± 0.02 mg GE/g dw). The highest loss was observed in the HTP group (49.76 ± 0.01%), followed by the AF group (25.26 ± 0.06%) and the microwaved group (12.37 ± 0.02%). Polysaccharides may be thermally degraded during heat treatment. High-pressure treatment can change the structure of macromolecules, therefore, HTP treatment may have affected the structural rearrangement of water-soluble polysaccharides in *UP*, thus reducing the extractability of polysaccharides [43]. The main reason for polysaccharide loss in the microwaved group could be the dissolution of polysaccharides in hot water [44].

#### 3.5.2. Chlorophyll a and Fucoxanthin

The HPLC diagram of chlorophyll a was shown in Figure S1. The chlorophyll a content is shown in Table 3. Different methods of cooking exerted different effects on chlorophyll a content in *UP*. Although reduction was observed with all cooking methods, The chlorophyll a loss was the highest at 51.01 ± 0.04% for the AF group, 25.61 ± 0.02% and 28.73 ± 0.10% for the microwaved and HTP groups, respectively. The significantly higher reduction in chlorophyll a by AF treatment can be attributed to the high temperature of 180 °C, which degraded chlorophyll a into pheophytin and other chlorophyll derivatives. The formation of pheophytin was accompanied by a change in color from bright green to olive brown [45]. During cooking, pheophytinization was the main reaction that affects chlorophyll a [46]. The acid released during processing could replace the magnesium in the porphyrin ring, and convert chlorophyll into pheophytin [47]. Since chlorophyll is stable under high pressure, the loss of chlorophyll a in the HTP group was also caused primarily by the high temperature heating. This result was consistent with that reported by Sánchez et al. [47]. The reason for the loss of chlorophyll a content in the microwaved samples may be due to the degradation of chlorophyll to pheophytin and the leaching of cell contents in boiling water [48].

Figure S2 showed the HPLC diagram of fucoxanthin. The contents of fucoxanthin in AF group, microwaved group and HTP group were 233.63 ± 12.21 μg/g dw, 281.78 ± 17.06 μg/g dw and 209.32 ± 8.48 μg/g dw, which were significantly reduced compared to the control group (335.20 ± 9.63 μg/g dw) (Table 3). The result in the microwaved group was consistent with Nie et al. [49]. Microwave treatment could destroy cell walls and subcellular structures, and led to release of fucoxanthin into boiling water. The structural characteristics of vegetable cell walls were different, which determined their ability to withstand heat treatment and to retain antioxidants [48]. The high-pressure environment in the electric pressure cooker increased the boiling point of water, thereby accelerating the degradation of fucoxanthin, which may be the cause of the loss observed in the HTP group. Susanto et al. [50] found that fucoxanthin in the methanol extract of cooked brown algae (*Sargassum ilicifolium*) was higher than that in the fresh sample. However, in this study, compared with uncooked sample, the fucoxanthin contents of all cooked samples were generally lower. In addition, the content of fucoxanthin measured in this experiment was less than that of Fung et al. [51], probably due to the hot-air drying used in this study. All these results suggested that fucoxanthin contents in foods could be affected by many factors, which must be taken into account when selecting appropriate cooking methods for food processing.

### 3.6. Amino Acids

Amino acids play an essential function in an organism [52], and they are the most important components of human tissues, enzymes and hormones [53]. For instance, the glutamic, arginine, leucine are important for health, growth and development and lactation [54]. Histidine and phenylalanine act as neurotransmitters, and threonine acts as a modulator of the immune system [55]. Table 4 lists the total amounts of all amino acids detected in this study for various samples. After microwave cooking, the loss of total amino acid content was the highest (16.81 ± 0.04%), followed by HTP (6.19 ± 0.06%) and AF (2.14 ± 0.15%). The decrease in total amino acid content was consistent with that reported by Guilherme C.L. Reis et al. [56]. However, the losses in this study were not significant (p > 0.05), which may be related to the difference in the research subjects and processing methods. The percentage of essential amino acids did not change significantly before and after cooking (control: 42.01 ± 0.07%; AF: 41.23 ± 0.06%; microwave: 41.21 ± 0.02%; HTP: 40.75 ± 0.03%). Glutamic acid, aspartic acid and alanine were the dominant amino acids, while the remaining amino acids were low in content. The dominant amino acids (glutamic acid, aspartic acid and alanine) accounted for 13.73 ± 0.02%, 10.90 ± 0.01% and 9.86 ± 0.01% in raw *UP*, 13.77 ± 0.02%, 11.58 ± 0.02% and 9.97 ± 0.01% in the AF group; 13.86 ± 0.01%, 11.56 ± 0.01% and 10.24 ± 0.01% in the microwaved group; and 13.91 ± 0.01%, 11.66 ± 0.01%, 10.13 ± 0.01% in HTP group, respectively. Except for a significant decrease in the content of methionine, no significant changes were observed in the content of other amino acids after cooking (*p* > 0.05). The significant decrease in methionine was beneficial because it was considered bitter amino acid [57]. 

## 4. Conclusions

This study indicated that different home cooking methods exhibited diverse effects on the quality, nutrients and bio-functional ingredients of *UP*. The effects of cooking methods on bioactive substances (total phenols, polysaccharide, chlorophyll a, and fucoxanthin) was important for enhancing the health benefits of cooked products to consumers. The texture is a kind of quality closely related to the overall acceptability of food to people. Therefore, it is particularly important to select appropriate home cooking methods to reduce the loss of nutrients, quality and bioactive components. The results showed that compared to AF and HTP, microwave cooking was able to retain the color and active small molecules that were beneficial to human health better, and produced a softer texture. However, it produced a higher loss of total amino acids in the cooked product. Taking everything into account, microwaving cooking appears to be the method of choice for processing *UP* to maintain most of its health benefits.

## Figures and Tables

**Table 1:** Effect of different types of domestic cooking techniques on color properties of *Undaria pinnatifida* (*UP)*.

Color Properties	Raw	AF	Microwave	HTP
L *	26.48 ± 1.63 ^a^	25.33 ± 1.38 ^a^	26.91 ± 1.09 ^a^	25.33 ± 1.30 ^a^
A *	−10.63 ± 0.20 ^a^	−7.54 ± 0.35 ^c^	−8.22 ± 0.68 ^b^	−6.76 ± 0.29 ^d^
B *	44.88 ± 2.64 ^a^	43.05 ± 2.49 ^a^	45.58 ± 1.82 ^a^	43.03 ± 3.81 ^a^
∆E	—	4.29 ± 1.49 ^b^	3.20 ± 0.66 ^b^	5.90 ± 1.37 ^a^
Samples color	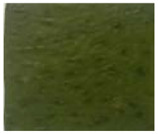	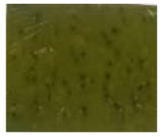	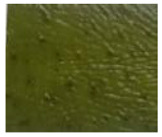	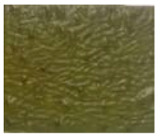

Data presented as mean and standard deviation (*n* = 6). Different letters in the same. row for different cooking methods indicate significant differences at (*p* ≤ 0.05). AF—air frying; HTP—high temperature and pressure.

**Table 2:** Texture parameters of *UP* under different cooking methods.

Cooking Methods	Hardness	Springiness	Cohesiveness	Chewiness	Resilience
Raw	702.02 ± 22.07 ^a^	39.11 ± 4.23 ^a^	0.81 ± 0.06 ^a^	22,450.92 ± 3717.30 ^a^	0.80 ± 0.05 ^a^
AF	520.52 ± 53.17 ^b^	30.43 ± 0.43 ^c^	0.79 ± 0.03 ^a^	12,510.89 ± 1515.66 ^c^	0.71 ± 0.08 ^b^
Microwave	294.61 ± 49.87 ^c^	21.21 ± 0.33 ^d^	0.68 ± 0.03 ^b^	4248.44 ± 873.74 ^d^	0.49 ± 0.06 ^c^
HTP	522.46 ± 24.26 ^b^	35.31 ± 2.70 ^b^	0.85 ± 0.05 ^a^	15,899.57 ± 2309.78 ^b^	0.76 ± 0.05 ^ab^

Data presented as mean and standard deviation (*n* = 5). Different letters in the same column for different cooking methods indicate significant differences at (*p* ≤ 0.05).

**Table 3:** Total phenol content (TPC), polysaccharide, chlorophyll a and fucoxanthin content of different cooking methods.

Cooking Methods	Total Phenols (mg GAE/g dw)	Polysaccharide (mg GE/g dw)	Chlorophyll a (mg/g fw)	Fucoxanthin (μg/g dw)
Raw	1.61 ± 0.0074 ^b^	4.76 ± 0.20 ^a^	24.32 ± 0.10 ^a^	335.20 ± 9.63 ^a^
AF	1.44 ± 0.0038 ^d^	3.56 ± 0.27 ^c^	11.98 ± 1.02 ^c^	233.63 ± 12.21 ^c^
Microwave	1.50 ± 0.0062 ^c^	4.17 ± 0.07 ^b^	18.18 ± 0.41 ^b^	281.78 ± 17.06 ^b^
HTP	1.69 ± 0.0075 ^a^	2.39 ± 0.02 ^d^	17.42 ± 2.43 ^b^	209.32 ± 8.48 ^d^

Data presented as mean and standard deviation (*n* = 3); dw: dry weight, fw: fresh weight. Different letters in the same column for different cooking methods indicate significant differences at (*p* ≤ 0.05).

**Table 4:** Total amino acid content of *UP* in different cooking methods.

Cooking Methods	Raw (mg/g dw)	AF (mg/g dw)	Microwave (mg/g dw)	HTP (mg/g dw)
Asp	16.60 ± 1.96 ^a^	17.27 ± 2.61 ^a^	14.64 ± 0.68 ^a^	16.65 ± 1.02 ^a^
Thr *	9.66 ± 1.50 ^a^	9.51 ± 1.49 ^a^	8.08 ± 0.40 ^a^	9.30 ± 0.54 ^a^
Ser	8.64 ± 1.37 ^a^	8.53 ± 1.34 ^a^	7.22 ± 0.34 ^a^	8.25 ± 0.51 ^a^
Glu	20.91 ± 3.31 ^a^	20.53 ± 3.12 ^a^	17.56 ± 0.79 ^a^	19.88 ± 1.23 ^a^
Gly	9.02 ± 1.49 ^a^	8.74 ± 1.35 ^a^	7.43 ± 0.37 ^a^	8.40 ± 0.53 ^a^
Ala	15.02 ± 2.25 ^a^	14.86 ± 2.20 ^a^	12.97 ± 0.80 ^a^	14.47 ± 0.84 ^a^
Val *	11.51 ± 1.79 ^a^	11.13 ± 1.54 ^a^	10.17 ± 1.12 ^a^	10.76 ± 0.63 ^a^
Met *	4.74 ± 0.87 ^a^	4.15 ± 0.57 ^ab^	3.36 ± 0.61 ^b^	3.23 ± 0.47 ^b^
Lle *	6.87 ± 1.35 ^a^	6.50 ± 1.09 ^a^	5.41 ± 0.26 ^a^	6.19 ± 0.43 ^a^
Leu *	11.35 ± 2.03 ^a^	11.09 ± 1.90 ^a^	9.18 ± 0.47 ^a^	10.63 ± 0.74 ^a^
Tyr	5.60 ± 0.86 ^a^	5.57 ± 0.88 ^a^	4.58 ± 0.22 ^a^	5.42 ± 0.31 ^a^
Phe *	11.58 ± 1.79 ^a^	11.10 ± 1.45 ^a^	9.39 ± 0.34 ^a^	10.40 ± 0.66 ^a^
Lys *	8.27 ± 1.31 ^a^	7.96 ± 1.35 ^a^	6.62 ± 0.35 ^a^	7.70 ± 0.53 ^a^
His *	3.73 ± 0.57 ^a^	3.63 ± 0.52 ^a^	3.10 ± 0.13 ^a^	3.46 ± 0.20 ^a^
Arg	8.80 ± 1.44 ^a^	8.46 ± 1.39 ^a^	6.98 ± 0.31 ^a^	8.12 ± 0.53 ^a^
∑AA	152.30 ± 23.50 ^a^	149.03 ± 22.65 ^a^	126.70 ± 6.63 ^a^	142.87 ± 8.96 ^a^

Data presented as mean and standard deviation (*n* = 3); dw-dry weight. Different letters in the same row for different cooking methods indicate significant differences at (*p* ≤ 0.05). * represents essential amino acids.

## Data Availability

Not Applicable.

## Supplementary Materials

The following are available online at https://www.mdpi.com/article/10.3390/foods10112786/s1, Figure S1: Typical HPLC diagram of chlorophyll a in different cooking methods. (a) Raw; (b) AF; (c) Microwave; (d) HTP; Figure S2: Typical HPLC diagram of fucoxanthin in different cooking methods. (a) Raw; (b) AF; (c) Microwave; (d) HTP.
