# Supplementary material for: Influence of Domestic Cooking on Quality, Nutrients and Bioactive Substances of Undaria pinnatifida
Source: Foods. 2021 Nov 12;10(11):2786. doi: 10.3390/foods10112786 (PMC8619837; doi:10.3390/foods10112786)
Supplement: Supplementary file 1 [file foods-10-02786-s001.zip › foods-1435023-supplementary.pdf]

Firstly, we should state that only in the pre-experimental stage, we conducted the method of sensory evaluation.

Before cooking, salted *UP* samples were soaked in water until they fully stretched out, and then washed 3-4 times with deionized water to remove the salt from the surface. In this period, the experiments including raw *UP* material desalination by flowing water and salty taste were done. The desalination effect was tasted by students in laboratory. The raw *UP* material did not taste salty meaning that most of salt were removed.

Then, in the period of cooking condition screening, color, texture, odour and flavor of the cooked *UP* samples were simply evaluated by students and teachers (10 people in total) in our laboratory. Since the domestic cooking methods were to make a dish of *UP*, we invited students and teachers to show their own feeling but not give their scores to the cooked *UP*. After mutual discussion, we determined the optimal cooking time for each method as follows: air frying (A.F) at 180 °C for 10 min, microwave treatment at 1700 W for 2.5 min, and high temperature and pressure (H.T.P) group for 10 min. During the pre-experiment period, the aim was to ensure that the cooking conditions for each method were relatively optimal. We then chose this condition that was considered relatively acceptable to continue our further studies in the manuscript.

Therefore, we would like to say that the sensory evaluation of cooked *UP* in the pre-experiments was only for making sure the relatively acceptable and edible *UP* for people.

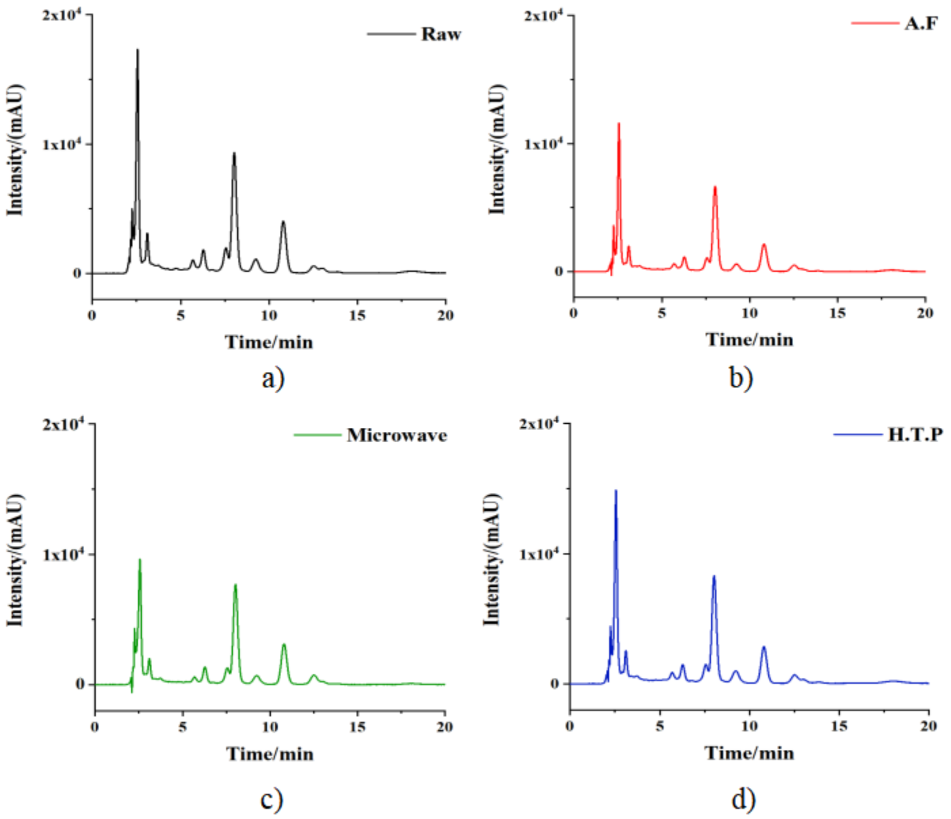

Figure S1. Typical HPLC diagram of chlorophyll a in different cooking methods. a) Raw; b) A.F; c) Microwave; d) H.T.P

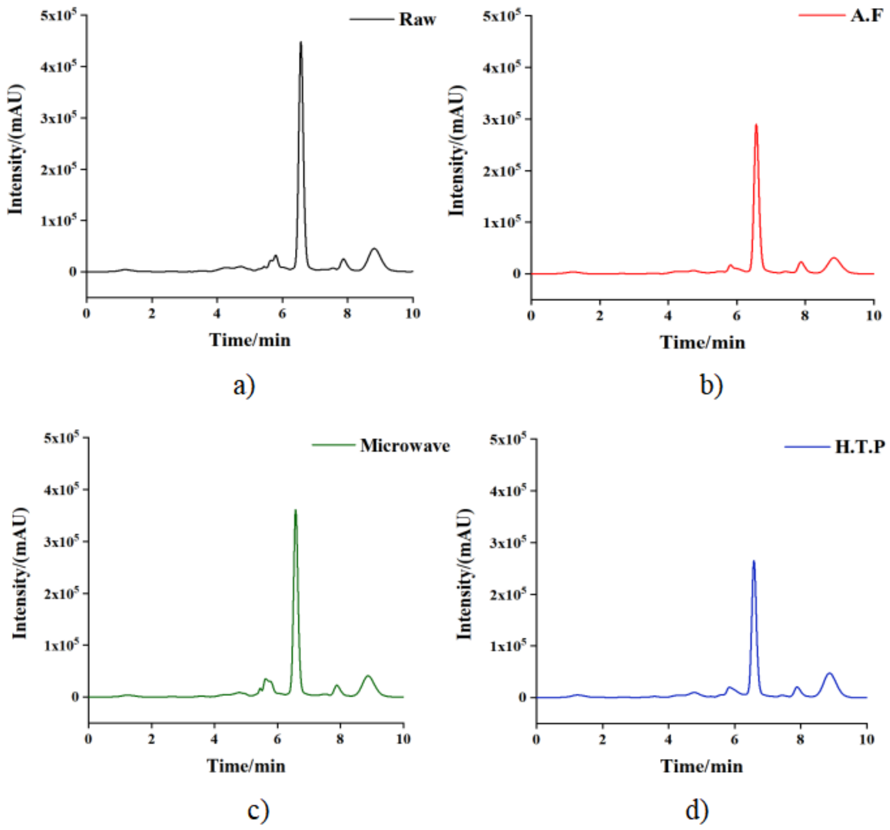

Figure S2. Typical HPLC diagram of fucoxanthin in different cooking methods. a) Raw; b) A.F; c) Microwave; d) H.T.P
